# Coumarin–Tetrapyrrolic Macrocycle Conjugates: Synthesis and Applications

**DOI:** 10.3390/molecules22060994

**Published:** 2017-06-15

**Authors:** Ana F. R. Cerqueira, Vítor A. S. Almodôvar, Maria G. P. M. S. Neves, Augusto C. Tomé

**Affiliations:** Department of Chemistry and QOPNA, University of Aveiro, 3810-193 Aveiro, Portugal; anacerqueira@ua.pt (A.F.R.C.); v.almodovar@ua.pt (V.A.S.A.); gneves@ua.pt (M.G.P.M.S.N.)

**Keywords:** coumarins, porphyrins, phthalocyanines, corroles

## Abstract

This review covers the synthesis of coumarin–porphyrin, coumarin–phthalocyanine and coumarin–corrole conjugates and their potential applications. While coumarin–phthalocyanine conjugates were obtained almost exclusively by tetramerization of coumarin-functionalized phthalonitriles, coumarin–porphyrin and coumarin–corrole conjugates were prepared by complementary approaches: (a) direct synthesis of the tetrapyrrolic macrocycle using formylcoumarins and pyrrole or (b) by functionalization of the tetrapyrrolic macrocycle. In the last approach a range of reaction types were used, namely 1,3-dipolar cycloadditions, hetero-Diels–Alder, Sonogashira, alkylation or acylation reactions. This is clearly a more versatile approach, leading to a larger diversity of conjugates and allowing the access to conjugates bearing one to up to 16 coumarin units.

## 1. Introduction

The coumarins are an important class of heterocyclic compounds with diverse pharmacological activities [1] and outstanding optical properties. Among their varied pharmacological activities, the anti-inflammatory [2], antibacterial [3,4], antiviral [5,6], and anti-cancer [7,8,9,10] properties can be highlighted. Due to their excellent optical properties, coumarin derivatives have been used in a variety of applications such as optical brighteners [11,12], optical sensors [13], organic light emitting diodes [14,15,16,17], laser dyes [18], photonic bandgap materials [19], light harvesting materials [20,21,22], and as fluorescent labels and probes in biology and medicine [23,24,25,26,27,28,29,30].

Due to their exceptional optical properties, coumarins are the focus of an intense research effort in various scientific areas. In particular, in recent years, a large number of coumarin–tetrapyrrolic macrocycle conjugates have been synthesized and their photophysical properties evaluated. Typically, the main objective of those studies is to take advantage from the efficient energy transfer between the coumarin and the tetrapyrrole unit, aiming for light harvesting applications. In this article, we review primarily the synthetic approaches leading to coumarin–porphyrin, coumarin–phthalocyanine, and coumarin–corrole conjugates. A range of other coumarin–chromophore conjugates, such as coumarin–bodipy [18,31], coumarin–fullerene [32,33,34], coumarin–perylene [35,36,37], etc. have also been reported and exhibit highly interesting photophysical properties. However, those systems are not covered in this review.

## 2. Coumarin–Tetrapyrrolic Macrocycle Conjugates

### 2.1. Coumarin–Porphyrin Dyads

**Scheme 1:**
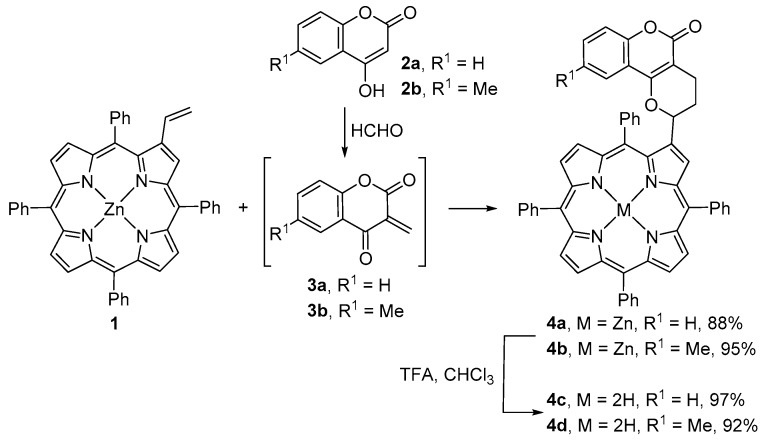
Route to coumarin–porphyrin dyads [38,39].

The number of porphyrin derivatives bearing coumarin units at β-pyrrolic positions are scarce. Examples of such type of compounds were reported in 2011 by Cavaleiro and co-workers [38,39]. The coumarin–porphyrin dyads **4a**,**b** were synthesized via hetero-Diels–Alder reaction of β-vinyl-*meso*-tetraphenylporphyrinatozinc(II) (**1**) [40] with the *ortho*-quinone methides **3a**,**b** generated in situ from the reaction of 4-hydroxycoumarins **2a**,**b** with paraformaldehyde (Scheme 1). The reactions were performed in refluxing 1,4-dioxane for one hour and the resulting dyads were isolated in 88% (**4a**) and 95% (**4b**) yield, respectively. The site and regioselectivity of these reactions are in agreement with those observed in similar systems. The sensing ability of these Zn(II) conjugates towards anions was evaluated by UV-vis and fluorescence measurements. Alterations in both absorption and emission spectra, or only in the emission spectra, were detected in the presence of Cl^−^, CN^−^ and CH_3_CO_2_^−^ [39]. The sensing ability of the free-base conjugates **4c** and **4d**, obtained in excellent yields by demetalation of the corresponding Zn(II) complexes with a mixture of TFA/CHCl_3_, was also evaluated, but in the presence of several metal ions [39]. Both dyads showed a colorimetric effect (a color change from purple to yellow) and an unprecedented selectivity for Hg(II), even in an EtOH/H_2_O mixture. The same colorimetric effect for Hg(II) was observed when dyad **4c** was incorporated in a cellulose support material. The same dyad, both in solution and in the solid support, also showed a colorimetric effect at different pH values.

The coumarin–porphyrin dyad **4a** showed to be a potential probe towards the alkaloids caffeine, nicotine and cotinine, with a stoichiometry of one alkaloid per ligand [41]. Additionally, it was evidenced that this probe can detect small amounts of cotinine (2.5 ± 0.3 μM) in dam water samples.

Conjugates **4a**–**4d**, and the corresponding Cu^2+^ and Ni^2+^ complexes, were studied by electrospray mass spectrometry (ESI-MS) and tandem mass spectrometry (MS/MS) [42]. It was observed that the main fragmentation mechanism occurs via a retro hetero-Diels–Alder pathway.

**Figure 1:**
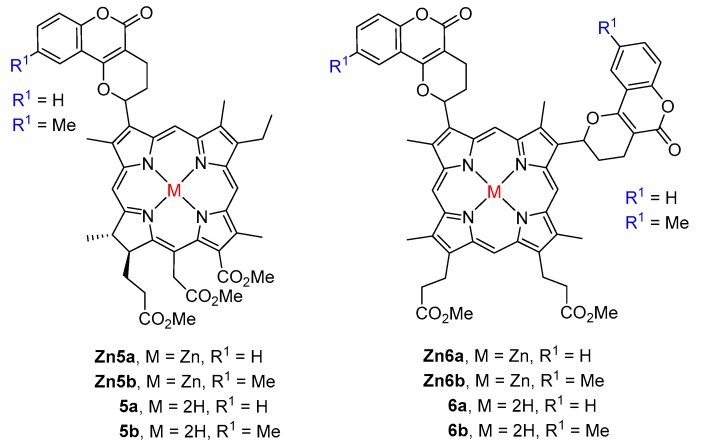
Structures of coumarin conjugates obtained from natural porphyrins [43].

The extension of the domino Knoevenagel/hetero-Diels–Alder approach to the zinc(II) complexes of chlorin e_6_ trimethyl ester and protoporphyrin IX dimethyl ester and the *ortho*-quinone methides **3a**,**b** afforded the conjugates **Zn5a**,**b** and **Zn6a**,**b** in excellent yields (81–91%) (Figure 1) [43]. These complexes were then demetalated to the corresponding free-bases **5a**,**b** and **6a**,**b**. These conjugates were isolated as mixtures of diastereomers and their relative abundances were determined by advanced NMR techniques. The evaluation of their photophysical and electrochemical properties showed that the fluorescence quantum yields of the chlorin e_6_ derivatives are higher than those of the protoporphyrin conjugates. All conjugates were able to generate singlet oxygen but protoporphyrin IX dyads gave the highest singlet oxygen quantum yields. The free-base chlorin e_6_ dyads showed an unexpectedly higher ability to generate singlet oxygen when compared with the Zn(II) counterparts due to the higher tendency of the complexes to aggregate. The same group reported the synthesis of six coumarin–porphyrin dyads **8a**–**f** from porphyrin **1** and the *ortho*-quinone methides **7a**–**f** generated in situ from 4-hydroxycoumarin and suitable aromatic aldehydes.

**Scheme 2:**
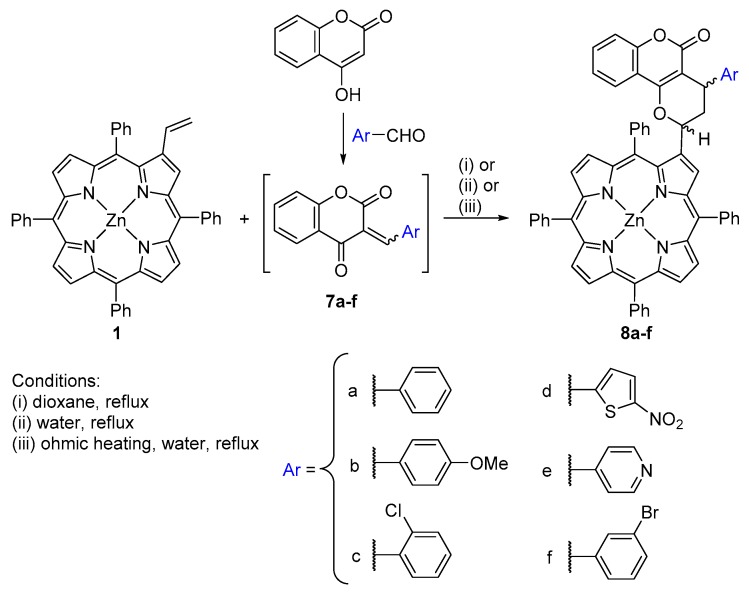
Synthesis of aryl-substituted coumarin–porphyrin dyads [44].

The reactions were carried out under three different conditions, including in water under ohmic heating (Scheme 2) [44]. Comparing the results obtained under the various experimental conditions, the authors concluded that the use of ohmic heating leads to a reduction of the reaction time, higher yields and selectivities. Moreover, the use of water as solvent facilitates the workup and product isolation over traditional organic solvents.

**Scheme 3:**
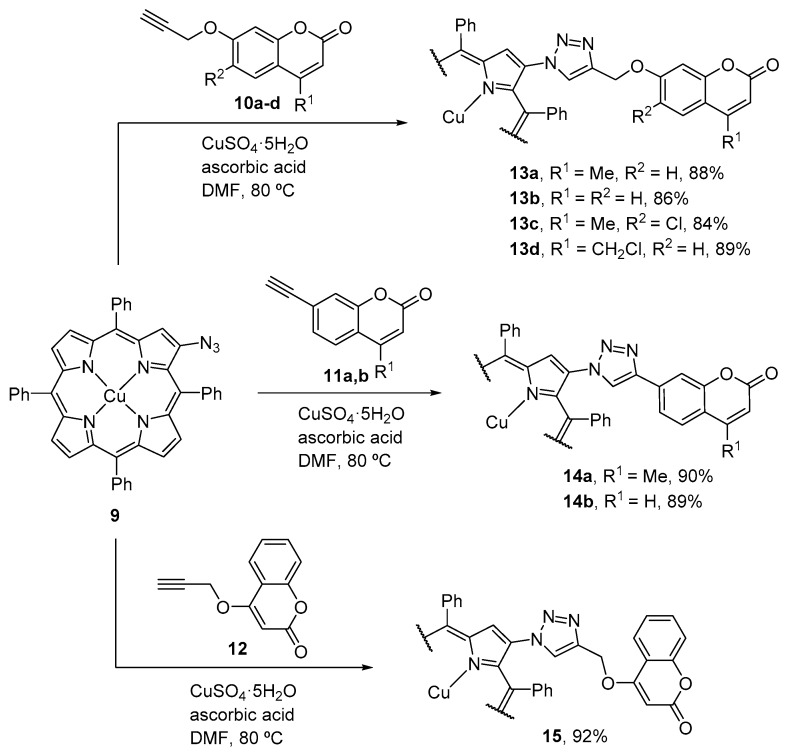
Synthesis of β-triazole bridged coumarin–Cu(II)porphyrin conjugates [46].

In 2015, Singh and Nath reported the synthesis of β-triazole bridged coumarin–porphyrin conjugates in 84–92% yield by the Cu(I)-catalyzed click reaction between 2-azido-5,10,15,20-tetraphenylporphyrinatocopper(II) (**9**) [45] with various alkyne-substituted coumarins **10**–**12** (Scheme 3) [46]. The authors also prepared the corresponding free-bases in good yield (71–80%) by demetalation of the copper derivatives. Metalation of the free-bases with zinc(II) acetate afforded the corresponding zinc(II) complexes in 91–96% yield. The photophysical characterization of these conjugates revealed, for some of them, a considerable electronic communication between both units. Additionally, in some of these dyads it was observed a significant intramolecular energy transfer between both chromophores.

**Scheme 4:**
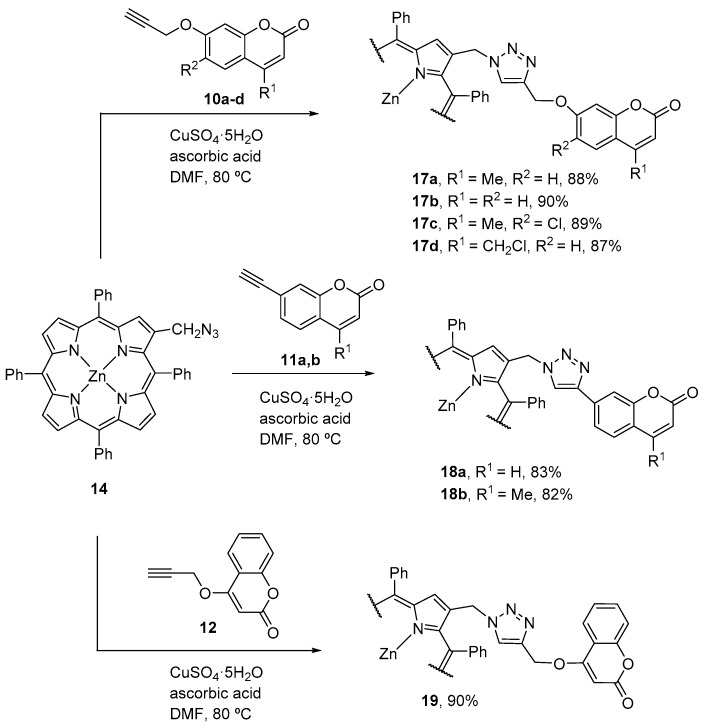
Synthesis of zinc(II) β-triazolylmethyl-bridged coumarin–porphyrin dyads [47].

Using a similar strategy, the same authors also described the synthesis of zinc(II) β-triazolylmethyl-bridged coumarin–porphyrin dyads **17**–**19** (Scheme 4) [47]. In these conjugates the porphyrin and the triazole units are separated by a methylene spacer. Their synthesis involved the 1,3-dipolar cycloaddition between the 2-azidomethyl-5,10,15,20-tetraphenylporphyrinatozinc(II) (**16**) and the alkyne-substituted coumarins **10**–**12**. The reported yields for the dyads are in the range 84–92%. Demetalation of the zinc(II) complexes afforded the corresponding dyads with free-base porphyrins in 69–79% yield. Again, the photophysical characterization of the new compounds allowed verifying an intramolecular energy transfer between both units for some of the conjugates.

**Scheme 5:**
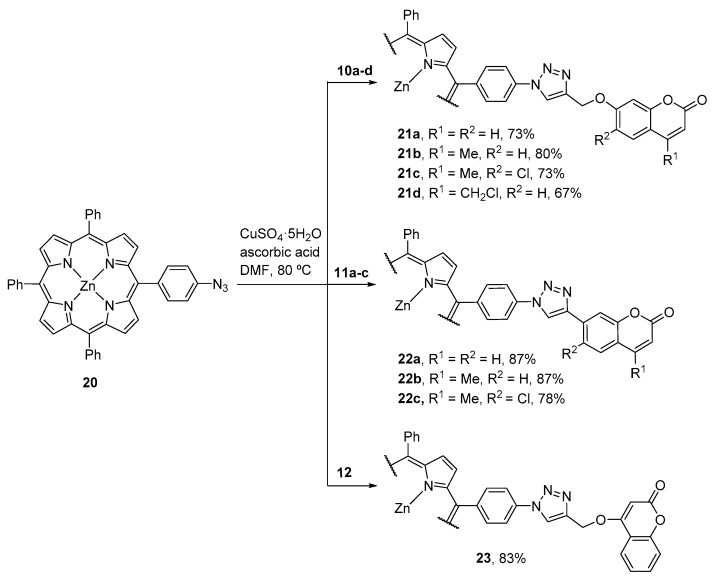
Synthesis of zinc (II) *meso*-phenyl-triazole bridged coumarin–porphyrin dyads [48].

The same alkyne-substituted coumarins **10**–**12** were used to prepare the zinc(II) *meso*-phenyl-triazole bridged coumarin–porphyrin dyads **21**–**23** through copper(I)-catalysed 1,3-dipolar cycloaddition reaction of zinc(II) 5-(4-azidophenyl)-10,15,20-triphenylporphyrin (**20**) (Scheme 5) [48]. The corresponding free-bases were successfully obtained after treatment with concentrated hydrochloric acid. The free-base dyads were converted into the nickel(II) complexes by treatment with nickel(II) acetate in chloroform–acetic acid. Preliminary photophysical results revealed a significant intramolecular energy transfer between both units for some of the zinc(II) conjugates.

**Figure 2:**
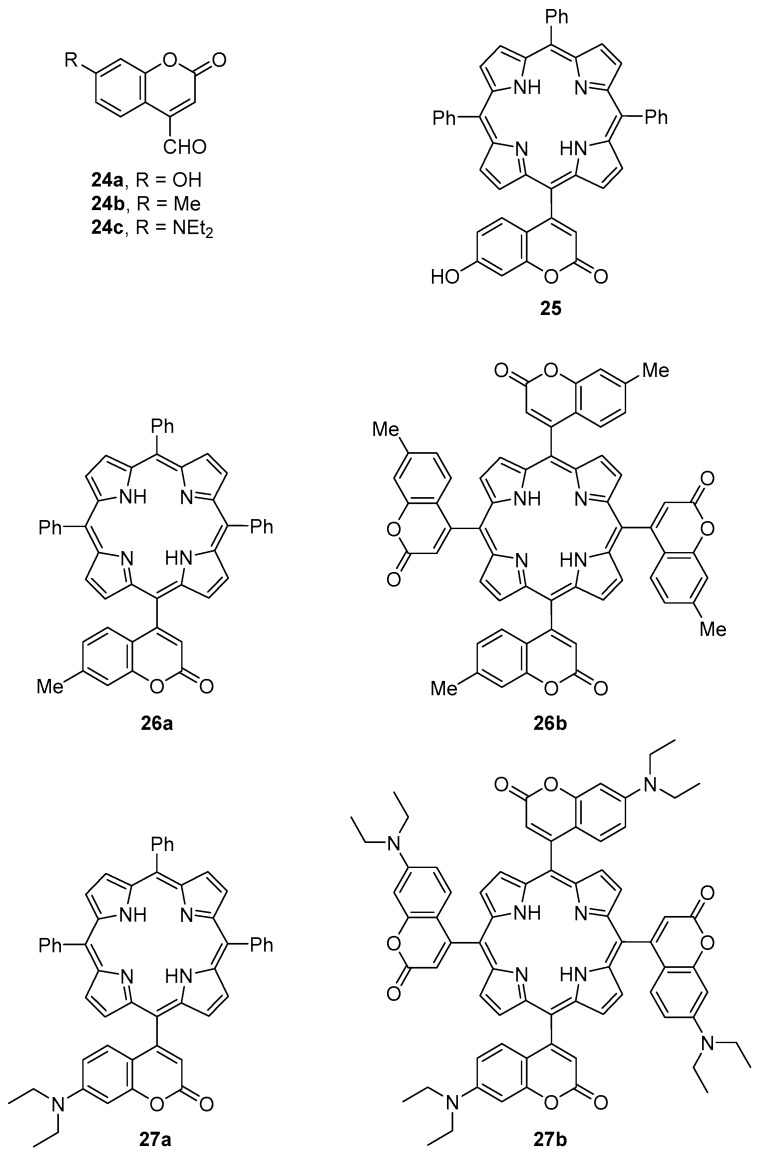
*meso*-(Coumarin-4-yl)porphyrins reported by Lin et al. [49].

Lin et al. reported the synthesis of symmetric and asymmetric porphyrins bearing coumarin units at the *meso* positions (Figure 2) [49]. The *mono*- and *meso*-tetrakis(coumarin-4-yl)porphyrins **25**–**27** were obtained in yields between ca. 5% and 13% from the reaction of pyrrole and an adequate molar ratio of benzaldehyde/coumarin-4-carbaldehydes **24a**–**c**. Depending on the coumarin−carbaldehyde used, the porphyrins where synthesized under Adler (refluxing propionic acid) [50] or Lindsey (BF_3_·OEt_2_ or TFA, CHCl_3_, room temperature, then *p*-chloranil) [51] conditions. The symmetrical *meso*-tetrakis(coumarin-4-yl)porphyrins **26b** and **27b**, isolated in 5.3% and 2.7% yield, respectively, were obtained from the reaction between pyrrole and coumarin−carbaldehydes **24b** and **24c** under Lindsey conditions. The absorption and photoluminescent spectra of these dyads in dilute THF solutions and as solid films (obtained by spin-coating the derivatives in quartz plates) demonstrated that the energy transfer from the coumarin substituents to the porphyrin core is more efficient in solid film than in solution. The best results were obtained with the dyads **27a**,**b** bearing the 7-diethylaminocoumarin moiety. This efficiency was justified by the high electron-donating ability of the amino substituent and also considering that in the solid state, the stacking porphyrins reduce the torsion angle between the porphyrin core and the coumarin substituent, forcing them to be coplanar and thus enhancing the conjugation extent of the porphyrin core and the coumarin substituent, facilitating the energy transfer from the coumarin units to the porphyrin core.

**Scheme 6:**
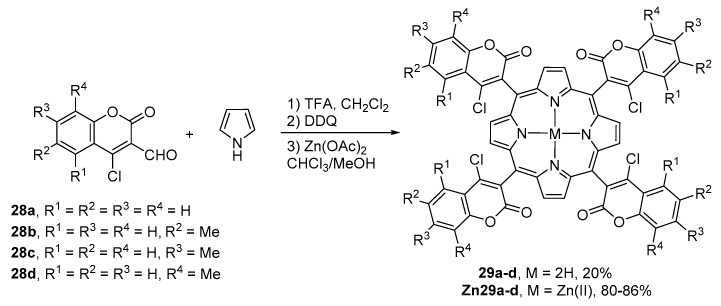
Route to *meso*-tetrakis(4-chlorocoumarin-3-yl)porphyrins reported by Amaravathi et al. [52].

The Lindsey experimental conditions were also used for the condensation of coumarin-3-carbaldehydes **28a**–**d** with pyrrole (Scheme 6) [52]. The resulting *meso*-tetrakis(4-chlorocoumarin-3-yl)porphyrins **29a**–**d** were obtained in ca. 20% yield as mixtures of four atropisomers. The authors were able to determine the ratio of the atropisomers by high-performance thin-layer chromatography (HPTLC) (UV-detector). The metalation of the free-bases with zinc(II) acetate in CHCl_3_–MeOH at room temperature afforded the corresponding zinc(II) complexes **Zn29a**–**d** in 80–86% yield.

**Scheme 7:**
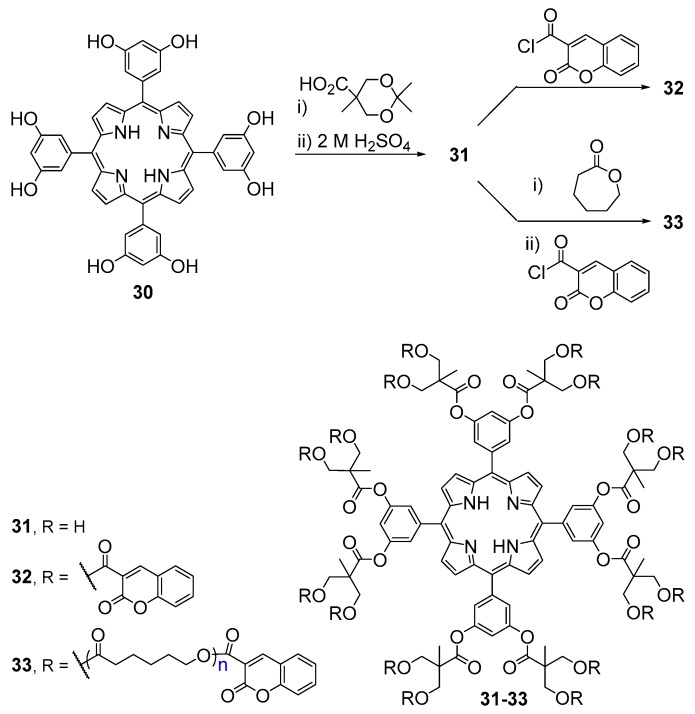
Route to coumarin–porphyrin conjugates reported by Fréchet and co-workers [53,54].

Fréchet and co-workers reported the synthesis of branched star polymers consisting of a central porphyrin core and 16 coumarin units (Scheme 7) [53,54,55]. The compounds were prepared via esterification of *meso*-tetrakis(3,5-dihydroxyphenyl)porphyrin (**30**) with acetonide-protected 2,2-bis(hydroxymethyl)propanoic acid followed by deprotection of the diol functionalities under acidic conditions. The reaction of the resulting hexadecahydroxy-functionalized porphyrin **31** with coumarin-3-carboxylic acid chloride gave the hexadecacoumarin-functionalized porphyrin **32**.

Bulk polymerization of ε-caprolactone with the initiator **31**, with tin(II) 2-ethylhexanoate as the catalyst, followed by esterification of the resulting polymer with coumarin-3-carboxylic acid chloride gave **33** in quantitative yield. Polymers **33** with varying chain lengths were prepared in almost quantitative yields by adjusting the monomer-to-initiator ratio. The 16 coumarin units in compounds **32** and **33** are responsible for the large absorption in the UV region of the spectrum. It was found that the presence of coumarin donor chromophores in these systems was particularly useful to evaluate the isolation of the core functionalities by using fluorescence resonance energy transfer (FRET). When the coumarin units were excited selectively at λ = 350 nm, the emission from both the coumarin donor and the porphyrin acceptor was observed demonstrating that in these systems FRET was facile but not quantitative. It was verified that as the chain length of the polymer increases the donor emission intensity increases as the result of the reduced probability of FRET. In compound **32**, due to the extremely short average donor–acceptor distance, a quantitative FRET was observed. Besides the chain length, it was verified that poor solvents seem to further increase the degree of site isolation due to a structural collapse of the polymer backbone giving rise to a more densely packed structure around the core unit and a reduced average donor–acceptor distance. This observation was supported by pulsed field gradient spin–echo (PGSE) NMR experiments that allowed the direct determination of the polymers molecular sizes in different solvents.

**Scheme 8:**
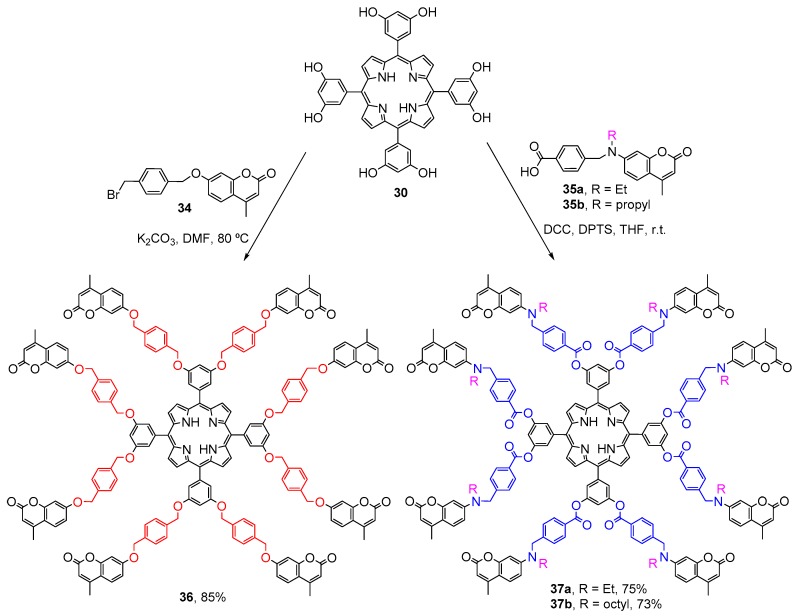
Synthesis of three porphyrin-core dendrimers with eight coumarin units [56].

Mao and Song also used *meso*-tetrakis(3,5-dihydroxyphenyl)porphyrin (**30**) as a platform to synthesize three porphyrin-core dendrimers bearing eight coumarin units (Scheme 8) [56]. Dendrimer **36** was prepared in 85% yield by reacting porphyrin **30** with the coumarin derivative **34**. Dendrimers **37a** and **37b** were synthesized in 73–75% yield by coupling porphyrin **30** with the carboxylic acid terminated coumarins **35a**,**b** in the presence of DCC (*N*,*N*′-dicyclohexylcarbodiimide) and DPTS (4-(dimethylamino)pyridinium *p*-toluenesulfonate). The study of the photophysical properties of the dendrimers in CH_2_Cl_2_ solutions and in thin neat films revealed an intramolecular energy transfer from the coumarin units to the porphyrin core. The best energy-transfer efficiencies were found for the dendrimers **37a** and **37b** due to a better spectral overlap between the emission spectrum of the coumarin units and the absorption spectrum of the porphyrin moiety than in dendrimer **36**. Comparing the optical properties of both dendrimers **37**, it was found that the energy-transfer efficiency is better for **37b** than **37a** in both solid film and solution, probably due to the presence of the longer alkyl side-chain which improves its solubility and consequently prevents the coumarins from self-quenching. All dendrimers emit red light with higher fluorescence quantum yields than the free-base porphyrin. It was remarked that these systems could be applied as light-harvesting antenna.

**Scheme 9:**
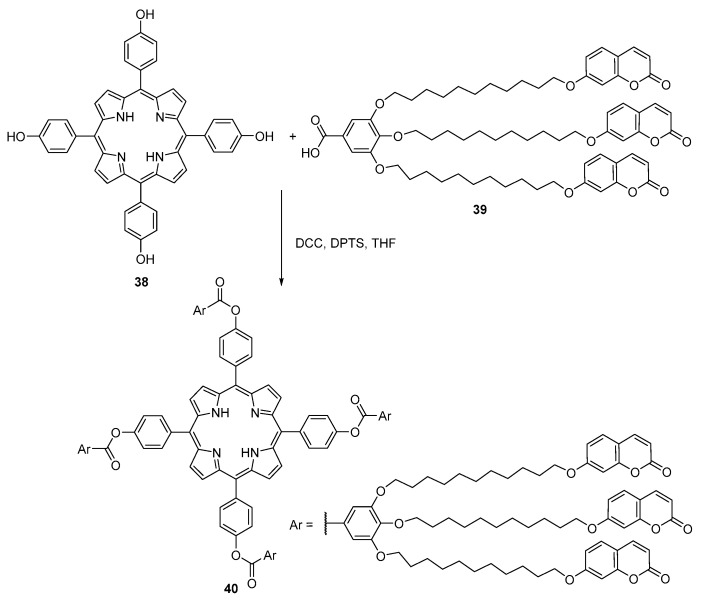
Synthesis of porphyrin-core dendrimers bearing 12 coumarin units [57].

Marcos and co-workers reported a new class of porphyrin-core dendrimers bearing 12 coumarin units via acylation of *meso*-tetrakis(4-hydroxyphenyl)porphyrin **38** with the coumarin-substituted benzoic acid derivative **39** (Scheme 9) [57]. The resulting compound **40** was obtained in 55% yield. Metalation of the free-base dendrimer **40** with zinc(II) acetate or with copper(II) acetate in CHCl_3_–MeOH afforded the corresponding zinc(II) or copper(II) complexes. All dendrimers formed nematic discotic mesophases and the charge mobility values of these materials are the highest ever reported for a nematic discotic phase. Excitation of the coumarin moieties (λ_exc_ = 320 nm) leaded to energy transfer to the porphyrin core. However, emission from both the coumarin units and the porphyrin acceptor was observed, thus demonstrating that FRET was facile but not quantitative in these systems [57].

**Scheme 10:**
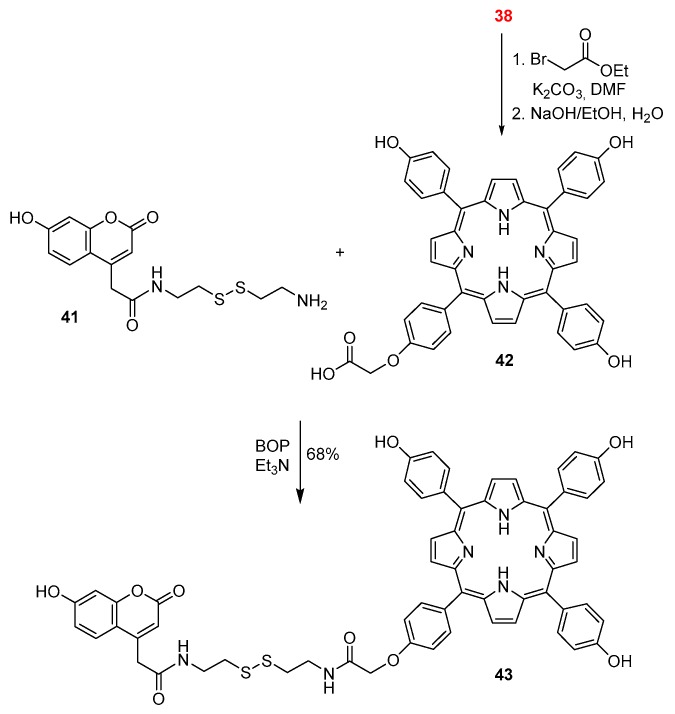
A coumarin–porphyrin dyad reported by Weiying Lin and co-workers [58].

Lin and co-workers also used *meso*-tetrakis(4-hydroxyphenyl)porphyrin (**38**) as a platform to synthesize a coumarin–porphyrin dyad (Scheme 10) [58]. This platform was alkylated with ethyl bromoacetate and the carboxylic acid **42** was obtained after hydrolysis of the ester group. The new dyad **43** was then prepared in 68% yield by coupling the amine-functionalized coumarin **41** with the carboxylic acid **42** in the presence of benzotriazol-1-yloxytris(dimethylamino)phosphonium hexafluorophosphate (BOP) and triethylamine. The dyad is highly selective and sensitive to thiols, exhibiting a remarkable change in emission colour from red to blue, being suitable for ratiometric fluorescence imaging of thiols in living cells.

**Scheme 11:**
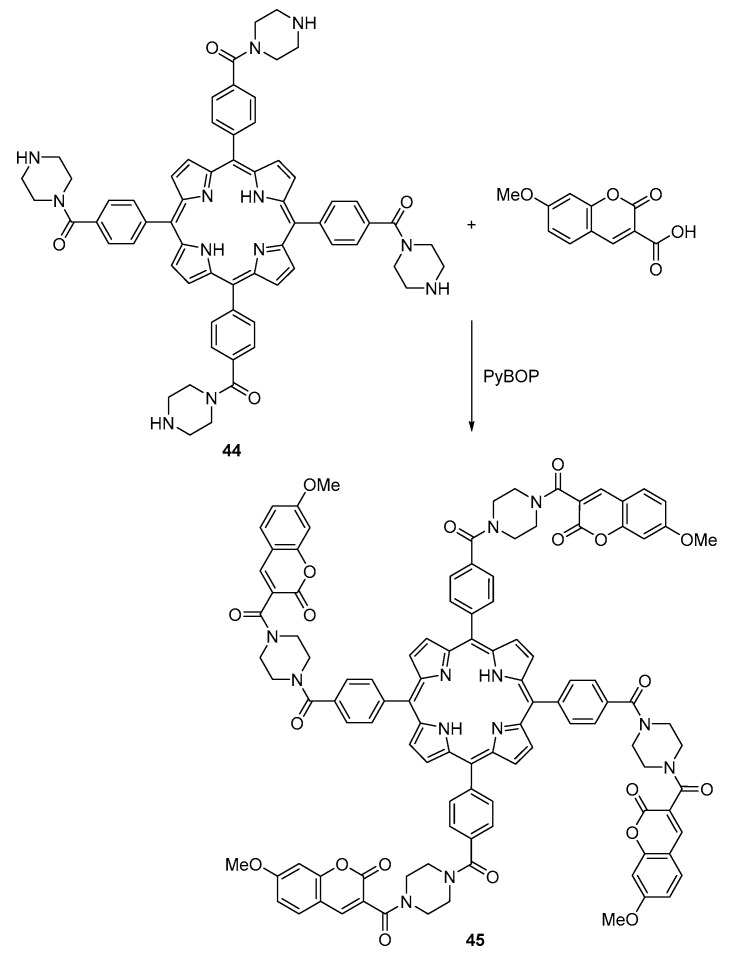
Synthesis of a donor–acceptor system [60].

A G3 dendrimeric coumarin-porphyrin conjugate consisting of a central Pt *meso*-tetrakis-(4-alkoxyphenyl)porphyrin linked to several coumarin-343 units was prepared and used as a probe for cellular two-photon oxygen imaging [59]. Knoester and co-workers reported the synthesis of a first-generation coumarin–porphyrin dendrimer where the porphyrin core is a derivative of *meso*-tetrakis(4-carboxyphenyl)porphyrin (Scheme 11) [60]. For the synthesis of this donor–acceptor system, *meso*-tetrakis(4-carboxyphenyl)porphyrin was first coupled to piperazine, using pivaloyl chloride as a coupling reagent, to form the piperazine-functionalized porphyrin **44**. The coumarin units were subsequently coupled to this porphyrin using (benzotriazol-1-yloxy)tripyrrolidin-1-yl)phosphonium hexafluorophosphate (PyBOP, a peptide coupling reagent), to give compound **45**. The energy transfer kinetics (from coumarin to porphyrin) was shown to be fast (transfer time ca. 500 fs) and efficient (transfer efficiency ca. 97%).

**Scheme 12:**
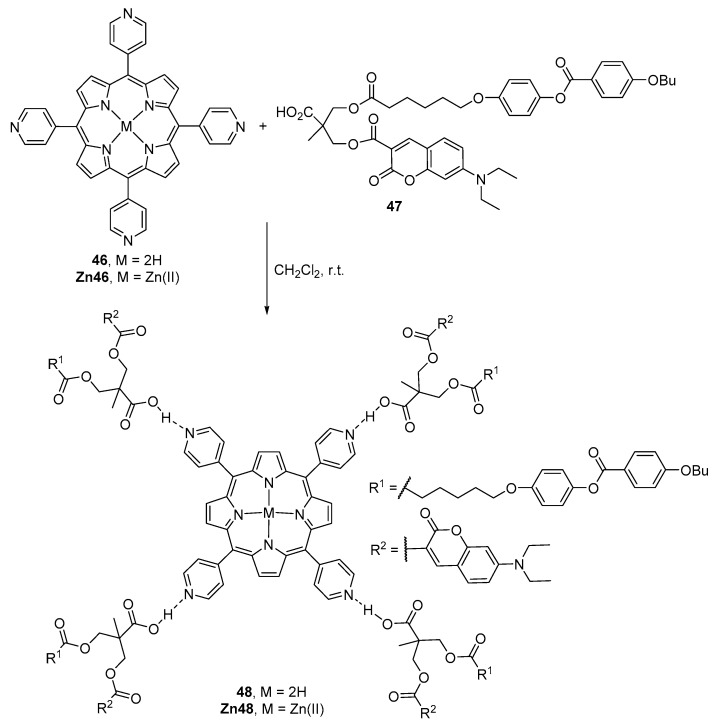
Synthesis of supramolecular porphyrin dendrimers with coumarin units [61].

Marcos and co-workers reported the formation of supramolecular system based on hydrogen-bonding between a porphyrin core and carboxylic acid dendrons functionalized with coumarin units (Scheme 12) [61]. The supramolecular porphyrin–coumarin dendrimers were prepared by mixing a dichloromethane solution of *meso*-tetra(4-pyridyl)porphyrin (**46**), or its Zn(II) complex (**Zn46**), and four equivalents of the carboxylic acid **47**. The slow evaporation of the solvent by stirring at room temperature afforded the dendrimers **48** and **Zn48**. Their stoichiometry in the condensed phase is 1:4, as corroborated by NMR spectroscopy. These supramolecular complexes do not show liquid crystalline behaviour and their absorption and emission spectra are a combination of the spectra of the corresponding building blocks. The lack of energy transfer between the two chromophores is probably due to the long distance between them.

**Scheme 13:**
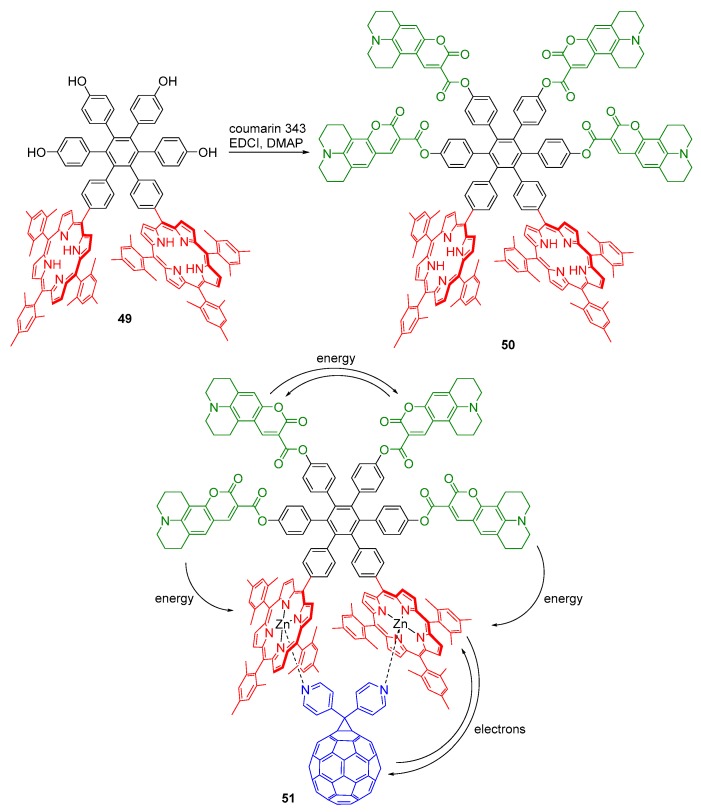
Synthesis of an artificial photosynthetic reaction center [62].

Gust and co-workers reported the synthesis of a molecular hexad **50** comprising two porphyrin moieties and four coumarin antenna chromophores, all organized by a central hexaphenylbenzene core (Scheme 13) [62]. The metalated hexad self-assembles with a pyridyl-bearing fullerene moiety, through coordination with the zinc atoms of the porphyrins, to yield the artificial photosynthetic reaction center **51**.

It was shown that light absorbed by any of the coumarins in hexad **50** is transferred to a porphyrin on the 1–10 ps time scale, depending on the site of initial excitation. The quantum yield of singlet energy transfer is 1.0. In heptad **51**, energy transfer to the porphyrins is followed by photoinduced electron transfer to the fullerene, resulting in a charge-separated state (P^•+^–C_60_^•−^) with an overall quantum yield of 1.0 [62].

**Figure 3:**
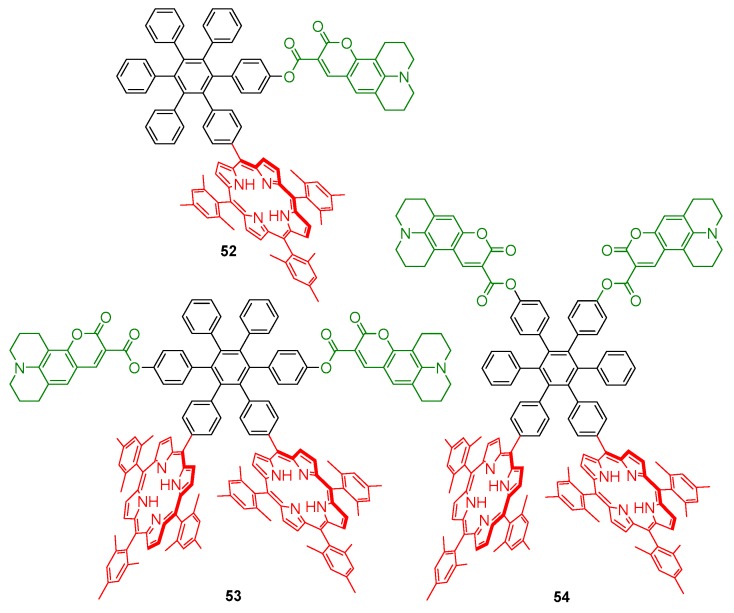
Model compounds of artificial photosynthetic reaction centers [62].

Compound **50** was prepared in 90% yield by coupling compound **49** with coumarin 343 using 1-ethyl-3-(3-dimethylaminopropyl)carbodiimide (EDCI) and 4-dimethylaminopyridine (DMAP). The synthesis of other compounds of type **50**, with one or two coumarin units (**52**–**54**, Figure 3), was also reported [62].

These studies allowed to conclude that in spite of the relatively long, flexible ester linkages between the coumarins and the hexaphenylbenzene ring, coumarin moieties are well suited as antennas in the 400−460 nm spectral range for porphyrin based artificial photosynthetic reaction centers. The high transfer rates, and therefore efficiencies, are ensured by coumarin singlet excited state lifetimes of several ns and high fluorescence quantum yields.

### 2.2. Coumarin–Phthalocyanine Dyads

**Scheme 14:**
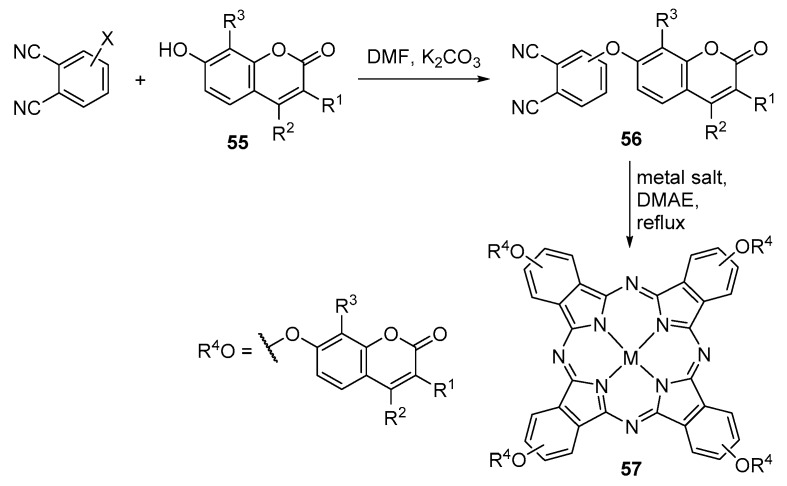
Typical route to coumarin–phthalocyanine conjugates [63,64,65,66,67,68,69,70,71,72,73,74,75,76,77,78,79,80,81,82,83,84,85,86,87,88,89,90,91].

Several coumarin–phthalocyanine conjugates have been reported in the last years. Many of those conjugates were synthesized from substituted 7-hydroxycoumarins **55** following the synthetic route shown in Scheme 14. Typically the first step involves the formation of a coumarin-substituted phthalonitrile, usually by nucleophilic displacement of a nitro group in 3- or 4-nitrophthalonitrile, and the resulting phthalonitriles **56** are then converted into metallophthalocyanines **57** by cyclotetramerization in 2-(dimethylamino)ethanol in the presence of a metal salt. The coumarins and phthalonitriles used in many of these reactions, and the metals in the phthalocyanine complexes, are shown in Table 1 [63,64,65,66,67,68,69,70,71,72,73,74,75,76,77,78,79,80,81,82,83,84,85,86,87,88,89,90,91].

**Scheme 15:**
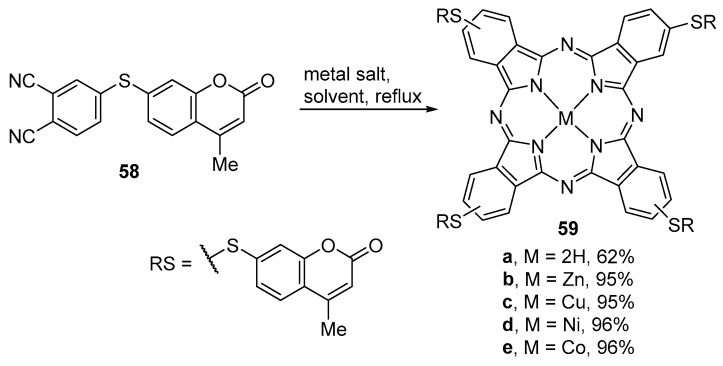
Synthesis of metal-free and metallophthalocyanines [64].

The method described above was also used for the synthesis of phthalocyanines bearing four peripheral 7-thio-coumarin units. The metal-free phthalocyanine **59a** and the metallophthalocyanines **59b**–**d** (Scheme 15) were prepared in high yields from the phthalonitrile derivative **58**, respectively in 2-dimethylaminoethanol (DMAE) at 145 °C or in quinoline at 195 °C, in a sealed tube [64].

**Scheme 16:**
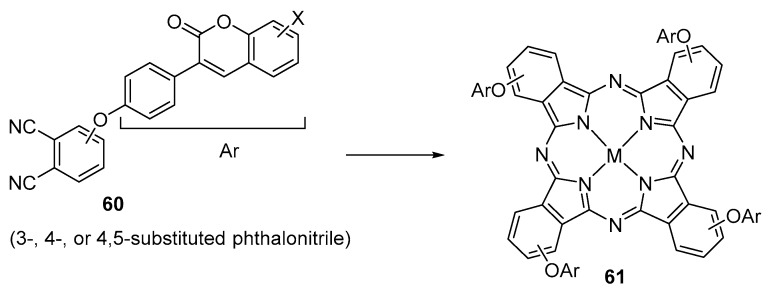
Synthesis of coumarin–phthalocyanine conjugates **61** [92,93,94,95,96,97,98,99,100,101,102,103,104].

The group of Bulut reported the synthesis of several phthalonitriles **60** and their conversion into phthalocyanines **61** carrying four or eight 4-(coumarin-3-yl)phenoxy substituents (Scheme 16 and Table 2) [92,93,94,95,96,97,98,99,100,101,102,103,104].

**Scheme 17:**
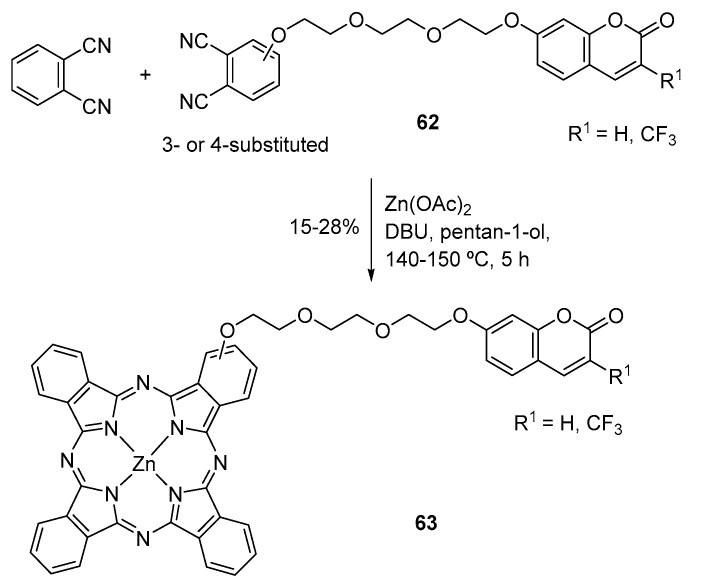
Synthesis of coumarin–phthalocyanine conjugates [105].

A recent study report the preparation of four coumarin–phthalocyanine conjugates bearing only one coumarin unit in which the coumarin moiety is linked to the phthalocyanine core at position 1 or 2 via a tri(ethylene glycol) spacer (**63**, Scheme 17) [105]. These conjugates were evaluated as potential anticancer agents with dual photodynamic and chemotherapy activity. Upon illumination at 670 nm, they show substantial cytotoxicity against HepG2 human hepatocarcinoma cells, with IC_50_ values in the range of 0.014–0.044 µM. The conjugate bearing the coumarin substituent at position 1 of the phthalocyanine (and R^1^ = H) exhibits high photocytotoxicity and significant chemocytotoxicity (IC_50_ = 4.43 µM). The results indicate that the coumarin and the phthalocyanine components of this conjugate work in a cooperative fashion [105].

**Scheme 18:**
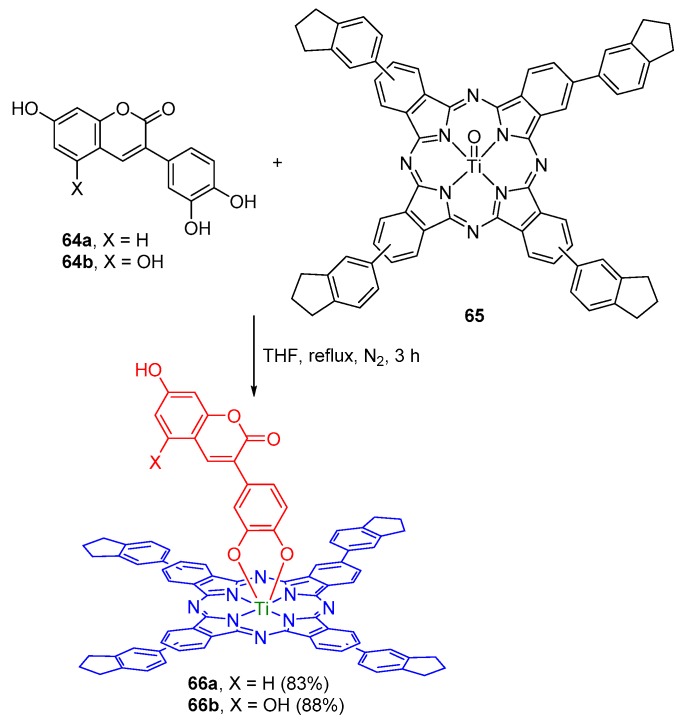
Synthesis of axial coumarin-substituted titanium(IV) phthalocyanines [106].

Bulut and co-workers reported the synthesis of the axial coumarin-substituted titanium(IV) phthalocyanines **66a**,**b** and their characterization by IR, UV–Vis, fluorescence, ^1^H-NMR, and MALDI-TOF MS [106]. The new conjugates were obtained in high yields from the reaction of the *ortho*-dihydroxyphenylcoumarin derivatives **64a**,**b** with the oxotitanium(IV) phthalocyanine **65** (Scheme 18). The UV–Vis spectra revealed small red-shifts of the Q-bands of the conjugates **66a**,**b** when compared to the Q-band of the oxotitanium(IV) phthalocyanine **65**. They also suggested that the conjugates have low aggregation tendency in nonpolar solvents. The fluorescence studies showed that the emission intensity is diminished by the axial coumarin substituents. Metal–insulator–semiconductor capacitors incorporating spin coated films of phthalocyanines **65** and **66a**,**b** as the insulating layer showed a rectification behavior.

### 2.3. Coumarin–Corrole Conjugates

**Scheme 19:**
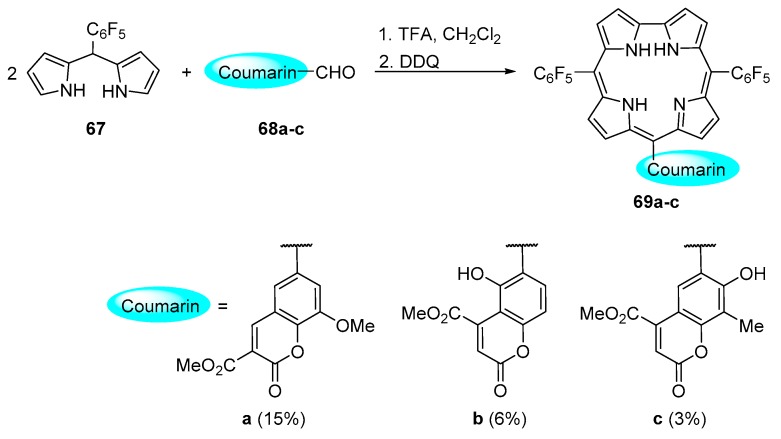
Coumarin–corrole dyads obtained by the [2 + 1] approach [108].

**Scheme 20:**
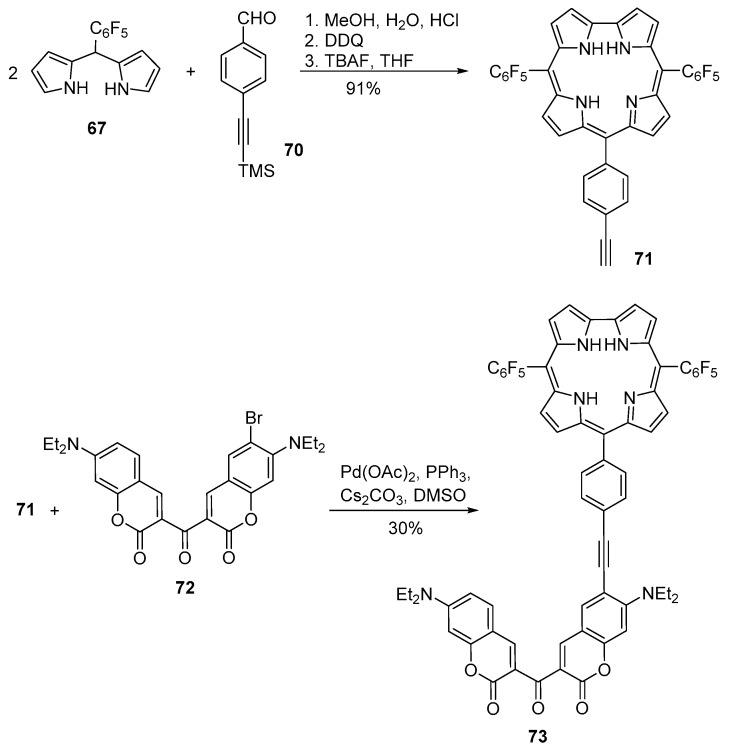
Coumarin–corrole dyad obtained under copper-free Sonogashira conditions [108].

Corroles display highly interesting and unusual photophysical and chemical properties. The methods for their functionalization were reviewed recently [107]. Despite the importance of such tetrapyrrolic compounds, published works on the synthesis of coumarin–corrole conjugates are scarce. In fact, the first synthesis of coumarin–corrole conjugates was reported only in 2010 by Gryko and co-workers [108]. These authors used two distinct approaches to synthesize the coumarin–corrole conjugates: (a) direct synthesis of the conjugates from the condensation of a dipyrromethane with a coumarincarbaldehyde (Scheme 19) or (b) functionalization of an ethynyl-substituted corrole with a bromocoumarin derivative via a Sonogashira reaction (Scheme 20).

The method used to prepare conjugates **69a**–**c**, that bear a direct link between both chromophores, involved the condensation of 5-(pentafluorophenyl)dipyrromethane **67** [109] with the coumarincarbaldehydes **68a**–**c** in CH_2_Cl_2_/trifluoroacetic acid (TFA) followed by oxidation with DDQ, according to established conditions [110]. The authors justified the relative low yields obtained in the [2 + 1] strategy for corroles **69a**–**c** due to a competitive Michael addition of the dipyrromethane to the coumarin α,β-unsaturated system.

The dyad **73** was synthesized by coupling the ethynyl-corrole **71** with the adequate bromo-ketobiscoumarin **72** under copper-free Sonogashira conditions (Scheme 20) [108]. Corrole **71** was obtained from the reaction of the TMS-protected ethynylbenzaldehyde **70** [111] with dipyrromethane **67** in aqueous methanol in the presence of HCl, followed by oxidation with DDQ [112] and then removal of the protecting group with tetrabutylammonium fluoride (TBAF).

Spectroscopic studies revealed that in all dyads the electronic coupling between the components is weak. In addition, there is a quantitative and extremely fast energy transfer from the coumarin moiety to the corrole unit [108]. This behaviour was justified considering the significant Stokes shift and the resulting overlap of coumarin emission with corrole absorption as well as the short spacer between the two units. The energy transfer was ascribed to a dipole–dipole mechanism. In dyad **69b** it was detected an electron-transfer from the excited corrole to the coumarin, yielding the low lying charge separated state Cum^−^–Corr^+^.

**Figure 4:**
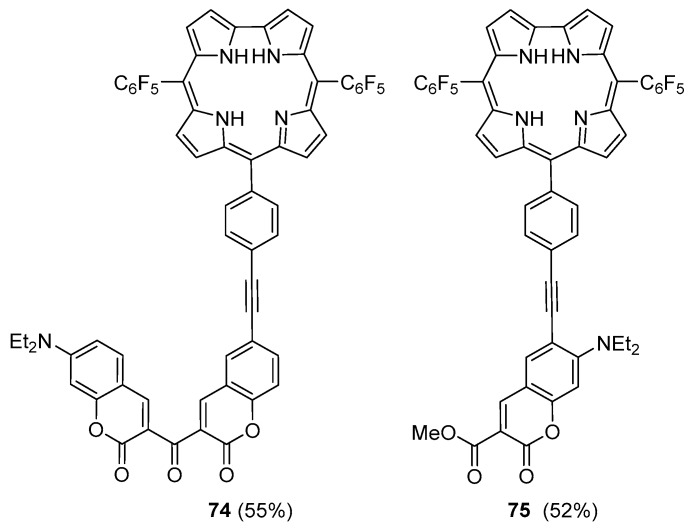
Coumarin–corrole dyads obtained under copper-free Sonogashira conditions [113].

The same group extended the copper-free Sonogashira approach to the synthesis of dyads **74** and **75** (Figure 4) [113]. The coupling involved the ethynyl-corrole **71** and the adequate 6-bromocoumarins. The cross-coupling reactions were performed in DMF in the presence of Pd(AcO)_2_, PPh_3_ and Cs_2_CO_3_ and the dyads were obtained in moderate yields.

**Figure 5:**
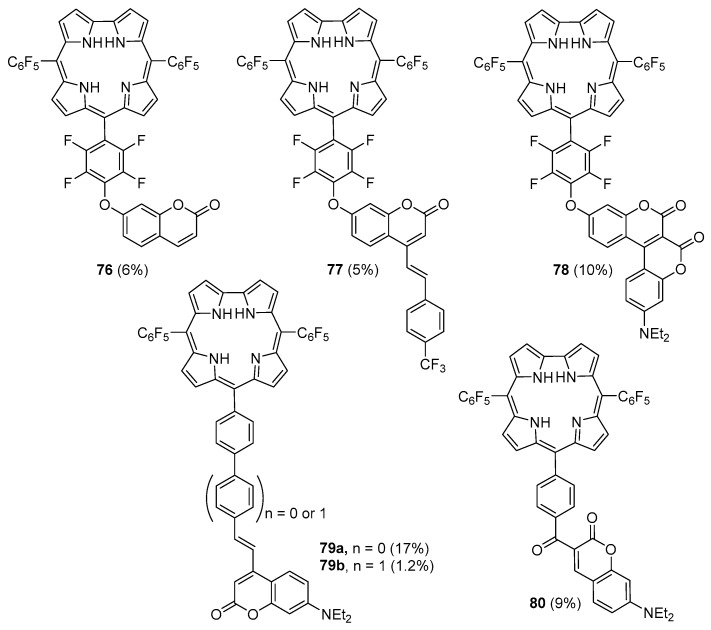
Coumarin–corrole dyads obtained by the [2 + 1] strategy [113].

In the same article it was also reported the synthesis of a series of coumarin–corrole dyads (**76**–**80**, Figure 5) via condensation of dipyrromethane **67** with suitable formylcoumarins [113]. These condensations were performed in CH_2_Cl_2_/trifluoroacetic acid followed by oxidation with DDQ and again the competitive Michael addition of the dipyrromethane to the α,β-unsaturated coumarins was considered responsible by the low yields of the desired dyads.

**Scheme 21:**
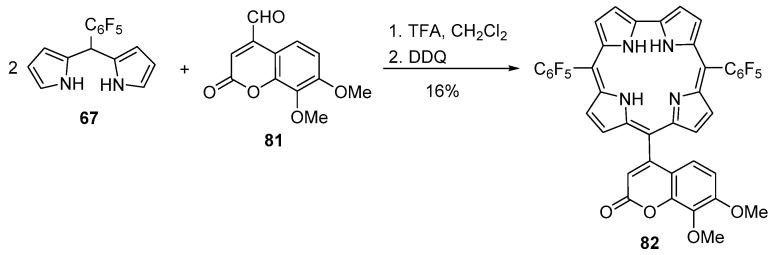
Synthesis of the dyad **82** [114].

Gryko, Wróbel and co-workers reported the synthesis of several *trans*-**A_2_B**-corroles, where **A** is the C_6_F_5_ group and **B** is an aryl or hetaryl group, and their use in a comparative study of their spectroscopic features [114]. One of those compounds was the coumarin–corrole **82** that was obtained from the reaction of dipyrromethane **67** with the coumarin-4-carbaldehyde **81** (Scheme 21). The yield obtained (16%) is much higher than the ones of related systems reported in previous studies (ca. 5–8%) [108,113]. This result was attributed to the much weaker polarization of 7,8-dimetoxycoumarin-4-carbaldehyde **81** and consequently the side reactions were minimized. Spectroscopic studies (absorption and excitation emission and fluorescence life-time values) of this dye in chloroform allowed to rule out the formation of aggregates even in highly concentrated solutions. The experimental data were supported by quantum chemical calculations (TD-DFT) of the HOMO and the LUMO. The electron spin resonance (ESR) studies before and after light illumination demonstrated that an unpaired electron is localized on the corrole core but not on the substituent.

**Scheme 22:**
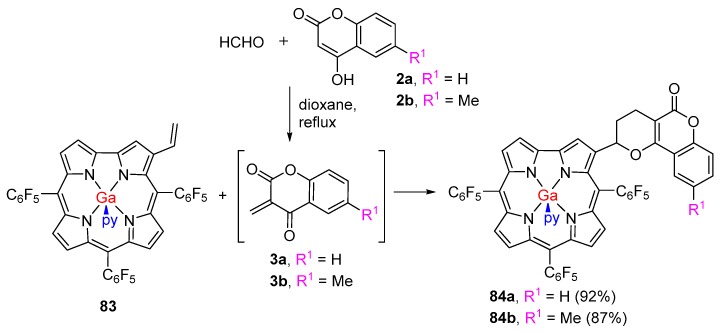
Synthesis of coumarin–corrole conjugates via hetero-Diels–Alder reactions [39].

Neves and co-workers developed a new strategy to prepare coumarin–corrole dyads where the coumarin unit is linked to a β-pyrrolic position of the corrole [39]. The new method involved a hetero-Diels–Alder reaction between the 3-vinylcorrole **83** [115] with *o*-quinone methides generated in situ from 4-hydroxycoumarins **2a**,**b** and paraformaldehyde (Scheme 22). The reactions were performed in refluxing dioxane and the cycloadducts **84a**,**b** were obtained in excellent yields.

**Figure 6:**
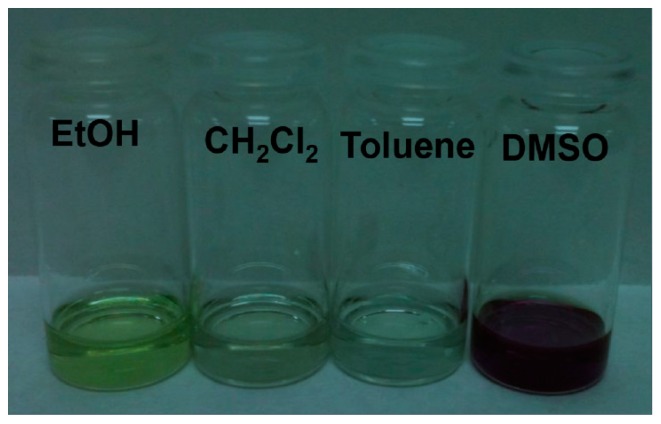
Solvatochromic effect of compound **84b**. Reprinted with permission from ref. [39]. Copyright © 2013 Elsevier Ltd.

The characterization of the photophysical properties of these dyads in various solvents (dichloromethane, DMSO, toluene, and ethanol) showed a strong solvatochromic effect for compound **84b**, easily detected by naked eye (Figure 6): a bathochromic shift of the absorption bands occurs with the increase of the solvent polarity. The sensorial ability of the coumarin–corrole dyads **84a**,**b** towards spherical (F^−^, Cl^−^), linear (CN^−^), and bulky (CH_3_COO^−^) anions was evaluated by UV-vis and fluorescence measurements and the more drastic alterations were detected for fluoride, cyanide and acetate.

## 3. Final Remarks

As shown in this review, several synthetic strategies aiming the synthesis of coumarin–tetrapyrrolic macrocycle conjugates were already developed. However, it is important to develop new and versatile synthetic routes to these compounds. In particular, the access to coumarin− phthalocyanine conjugates via post-functionalization of adequately substituted phthalocyanines deserves greater attention from synthetic chemists. Considering the well-known photophysical (and biological) properties of coumarins and tetrapyrrolic macrocycles, the corresponding conjugates are expected to be found in real applications in the coming years.

## Figures and Tables

**Table 1:** Starting materials and yields of coumarin–phthalocyanine conjugates **57**.

R^1^	R^2^	R^3^	X	M	57	Ref.
					Yield (%)	
H	H	H, Me	4-NO_2_	2H, Zn, Co, Ni, Cu	50–96	[63,64]
H	Me	H	-	2H, Zn, Co,	-	[65,66,67]
H	H, CF_3_	H	4-NO_2_	Zn, Co	51–58	[68]
H	4-MeOC_6_H_4_	Me	3-NO_2_, 4-NO_2_,	2H, Co, Zn, Fe	35–74	[69,70,71,72]
			4,5-dichloro			
H	CH_2_CO_2_H	Me	3-NO_2_	Zn, Co, In(OAc)	14–30	[73]
2-Cl-4-FC_6_H_3_	H	H	3-NO_2_, 4-NO_2_	2H, Co, Zn	16–36	[74,75]
3-MeOC_6_H_4_,	H	H	3-NO_2_, 4-NO_2_,	Zn, In, Mg, Mn	9–36	[76,77,78,79,80]
4-MeOC_6_H_4_,			4,5-dichloro			
3,4,5-(MeO)_3_C_6_H_2_						
3,5-F_2_C_6_H_3_	H	H	3-NO_2_, 4-NO_2_	Zn, In	38–39	[81]
biphenyl	H	H	3-NO_2_, 4-NO_2_	2H, In, Co, Zn	19–48	[82]
4-pyridyl	H	H	3-NO_2_, 4-NO_2_	Zn, Co	55–80	[83]
ferrocenyl	H	H	4-NO_2_	Zn, Co	45, 51	[84]
Me	Ph	H	3-NO_2_, 4-NO_2_,	In	26–31	[85,86]
			4,5-dichloro			
CO_2_Et	H	H	3-NO_2_, 4-NO_2_	Zn, Co, Ni, Cu	18–36	[87,88]
CH_2_CH_2_NEt_2_	Me	H	3-NO_2_, 4-NO_2_	2H, Zn	43, 77	[89,90,91]

**Table 2:** Coumarin–phthalocyanine conjugates **62** and yields.

Ar	Position of the ArO Group at the Phthalonitrile	M	61	Ref.
			Yield (%)	
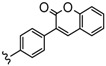	3 or 4	2H, Zn, Co, In(OAc)	15–50	[92]
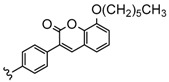	4	2H, Zn, Co	33–73	[93]
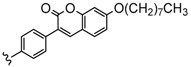	4	2H, Zn, Co, Ni, Cu	40–89	[94]
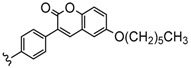	4	2H, Zn, Co	25–30	[95]
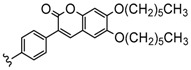	3 or 4	2H, Zn, Co, Cu	42–80	[96,97]
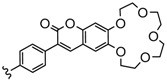	3 or 4	Zn, Co, Cu	34–53	[99,100,101]
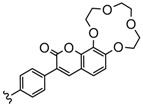	3 or 4	2H, Zn	23–68	[102]
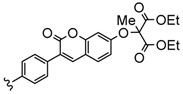	3 or 4	Zn, InCl	30–33	[103]
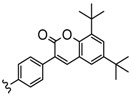	3 or 4 or 4,5	Zn, InOAc	40–57	[104]
